# Pseudoachalasia presenting 20 years after Nissen fundoplication: a case report

**DOI:** 10.1186/s13019-016-0495-y

**Published:** 2016-07-07

**Authors:** Chuong N. Lai, Kumar Krishnan, Min P. Kim, Brian J. Dunkin, Puja Gaur

**Affiliations:** Department of Medicine, Texas A&M University, College Station, TX USA; Department of Gastroenterology, Houston Methodist Hospital, Houston, TX USA; Department of General and Division of Thoracic Surgery, Weill Cornell Medical College, Houston Methodist Hospital, Houston, TX USA; Department of General Surgery, Houston Methodist Hospital, Houston, TX USA

**Keywords:** Pseudoachalasia, Prior Nissen, High-resolution manometry

## Abstract

**Background:**

Pseudoachalasia is a rare diagnosis manifested by clinical and physiologic symptoms of achalasia, with alternative etiology for outflow obstruction. While malignancy is a frequent cause of pseudoachalasia, prior surgical intervention especially surgery involving the esophagogastric junction, may result in a misdiagnosis of achalasia.

**Case presentation:**

We present a case of a 70 year-old male with dysphagia and weight loss after undergoing a Billroth I and Nissen fundoplication several decades ago. His preoperative studies suggested achalasia and he was therefore referred for an endoscopic myotomy. However, careful interpretation of all the data and intra-operative findings revealed a classic mechanical and functional obstruction requiring takedown of his prior wrap.

**Conclusions:**

Individualized interpretation of preoperative studies in the setting of prior foregut surgery is critical to appropriate diagnosis and intervention. This case highlights the significance of endoscopic findings and features of high-resolution manometry specific to pseudoachalasia, which contrasts with classical features of achalasia.

## Background

Achalasia is characterized by the absence of peristalsis along with impaired relaxation of the lower esophageal sphincter (LES) [1]. Clinically, this results in dilatation of the esophagus and symptoms of regurgitation and progressive dysphagia. Pseudoachalasia is a rare disease that has many clinical features similar to primary achalasia. It is frequently attributed to malignancies of the distal esophagus or gastric cardia [2]. These tumors result in chronic esophageal outflow obstruction that in turn result in muscular dysfunction of the esophageal body. Benign causes of pseudoachalasia include vascular obstruction (dysphagia aortica), submucosal tumors, congenital muscular rings of the distal esophagus, and mechanical obstruction from prior foregut surgeries. Usually, albeit not always, these patients develop dysphagia soon after their surgical intervention. In literature series, pseudoachalasia has been attributed to prior surgeries in about 12 % of the cases [2]. Here, we present a case of a patient with pseudoachalasia secondary to chronic esophagogastric junction (EGJ) outflow obstruction from prior Nissen fundoplication. The case illustrates the significance of recognizing some key features of pseudoachalasia on preoperative studies that differentiate it from achalasia, before committing patients to a surgical myotomy.

## Case presentation

**Fig. 1:**
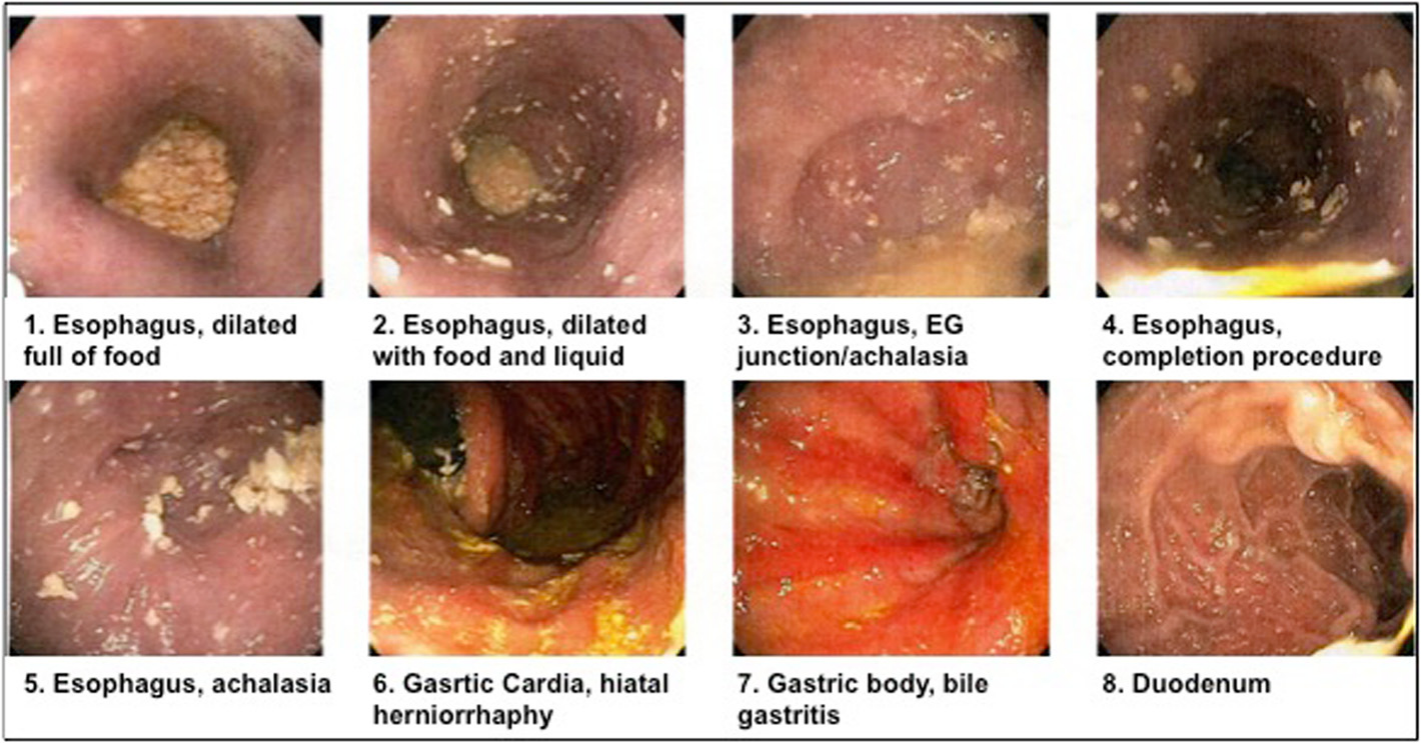
Endoscopy demonstrated markedly dilated esophagus with marked tortuosity. Esophagogastric junction was significantly narrowed and dilated. The report was read out as consistent with achalasia. Retroflex view (*lower panel*) demonstrated an intact fundoplication and prior gastrojejunal anastomosis was noted to be patent (image not shown)

A 70 year-old male with history of dysphagia and a 21 lb weight loss over 2–3 months was referred. At age 16, the patient had undergone repair of gastric ulcer perforation, followed by a Billroth I at age 28. In his late 40s, the patient underwent a Nissen fundoplication for symptomatic reflux disease (no preoperative outpatient records from his Nissen were available for review). He did well for 20 years and then developed progressive dysphagia. Endoscopy demonstrated a markedly dilated esophagus with tortuosity, full of liquids and solids (Fig. 1). The Nissen fundoplication was documented to be intact, and the narrowed EGJ was dilated to 20 mm with a hydrostatic balloon with modest relief. No documentation was made of a forced intubation of the LES. At this point, he was referred for an endoscopic myotomy for treatment of achalasia.

**Fig. 2:**
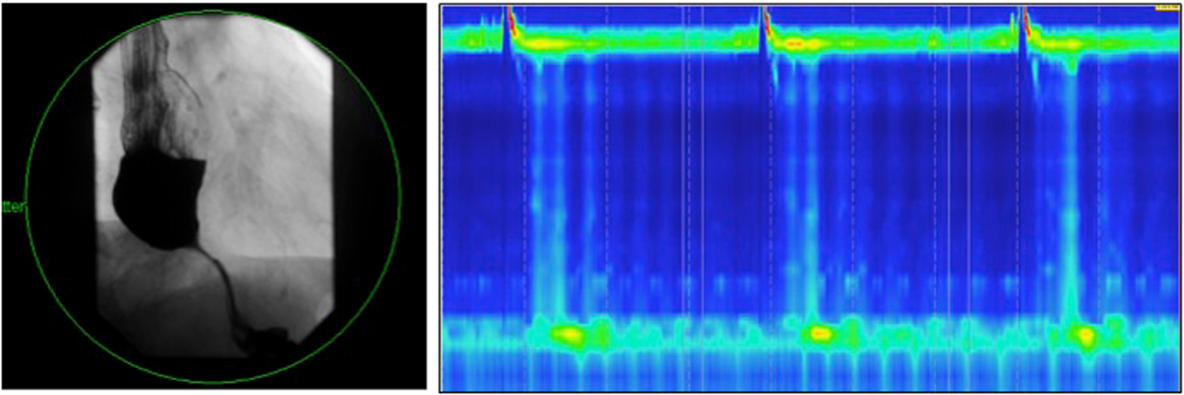
An esophagram prior to the operation demonstrated a narrowed esophagus as well as an intact Nissen wrap. The distal esophagus was significantly dilated. High-resolution manometry demonstrated 100 % failed swallows and the lower esophageal sphincter relaxed to gastric baseline

At our institution, the patient had an esophagram that demonstrated a fixed smooth narrowing in the region of the LES, concurring with a diagnosis of achalasia (Fig. 2). Manometry revealed 100 % aperistalsis, however the mean LES pressure was reported as normal (5.7 mm Hg). He further had a normal IRP (9 mm Hg) (Fig. 2). A chest CT scan did not show any evidence of extrinsic mass or aberrant vascular anatomy to explain his symptoms. After a thorough discussion regarding the case at our institutional foregut conference, it was determined that while the clinical and radiographic findings were consistent with achalasia, the lack of elevated IRP were inconsistent with impaired LES relaxation, and hence, it was felt that the etiology of outflow obstruction was more consistent with pseudoachalasia than from primary achalasia.

**Fig. 3:**
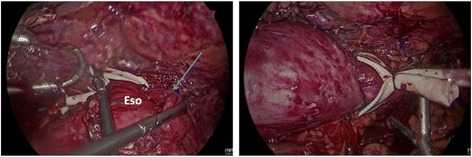
Intra-operatively, extensive lysis of adhesion was performed and the patient’s prior Nissen fundoplication was found to be partially undone. The *blue arrow* is pointing to the left limb of the wrapped stomach. After careful dissection, the left limb of the fundoplication was noted to be tethered around with a cord behind the esophagus resulting in significant esophageal torsion. Abbreviation: Eso = esophagus

At the beginning of the operation, an endoscopy was performed. The diagnostic gastroscope (Olympus H-190) met resistance at the level of the EGJ. It was eventually advanced and left in place. Laparoscopically, extensive lysis of adhesions was performed to identify the esophageal hiatus and the prior wrap. After careful dissection, it was noted that the patient’s Nissen fundoplication had become partially undone (Fig. 3, left panel). The left limb of the fundoplication was tethered posteriorly that resulted in a tight band. This led to tethering of the greater curvature of the stomach, swinging the entire esophagus to the right, resulting in torsion and forming a stricture at the distal esophagus (Fig. 3, right panel). After meticulous takedown of the old fundoplication, the endoscope easily passed through the LES. In order to prevent future reflux, a Dor fundoplication was performed and a jejunostomy feeding tube was placed given the patient’s nutrition status.

Postoperatively, the patient did well and did not have any complications. After obtaining a repeat esophagram on postoperative day 6 that did not demonstrate a leak, he was started on oral intake and weaned off his tubefeeds over the next month. One year later, the patient remains symptom free and has resumed a normal diet.

## Discussion

A small percentage of patients who undergo fundoplication develop chronic dysphagia. In most cases of dysphagia, it is due to postoperative edema, slipped or migrated fundoplication, or even disrupted fundoplication and it usually presents in the acute setting [3]. However, in some circumstances, prolonged mechanical outflow obstruction may lead to a burnt-out esophagus, thus mimicking a clinical picture of achalasia. It is important for clinicians to distinguish this phenomenon from primary achalasia.

**Fig. 4:**
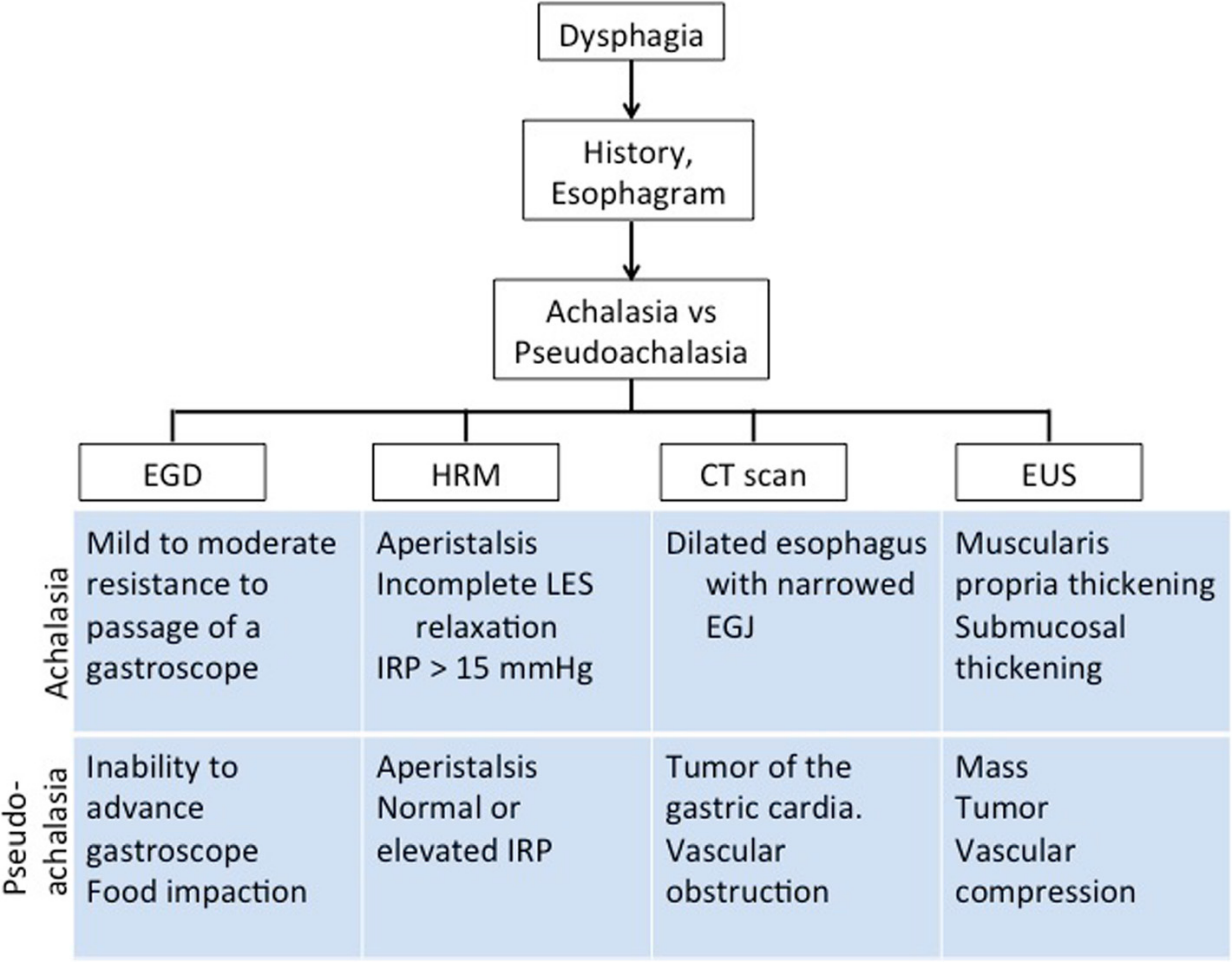
An algorithm that can be utilized to differentiate between achalasia and pseudoachalasia. Abbreviations: UGI, upper gastrointestinal series; EGD, esophagogastroduodenoscopy; HRM, high-resolution manometry; EUS, esophageal ultrasound; LES, lower esophageal sphincter; IRP, integrated relaxation pressure; EGJ, esophagogastric junction

Often a patient’s clinical presentation can help distinguish between the two clinical entities. While patients with achalasia tend to have long-duration symptomatology of years, patients with pseudoachalasia tend to have symptoms for only a few months prior to seeking medical care (primarily because of a malignancy that has a relatively more rapid-onset of dysphagia when compared to patients with achalasia) [2, 4]. Albeit, this is not universal, as you can see from our case who developed late-onset pseudoachalasia. In order to differentiate pseudoachalasia from achalasia, a few diagnostic tests can be performed to look for some key features of pseudoachalasia (Fig. 4). A barium study may suggest pseudoachalasia if the length of the narrowed segment is greater than 3.5 cm or the distal esophagus is asymmetric or nodular, instead of being smooth and asymmetric as in achalasia, although this is also rather non-specific [5]. Endoscopy is a good initial diagnostic tool to differentiate between the two conditions. Firm resistance or inability to pass a gastroscope through the EGJ should raise suspicion of pseudoachalasia [6, 7], which is often a result of too tight of a hiatal closure. Unlike conventional manometry, high-resolution manometry has become an efficient modality in evaluating esophageal motor disorders. Distinct manometric patterns such as EGJ outflow obstruction and normal integrated relaxation pressures also warrant further evaluation for alternative etiology [8]. A CT scan with 3D reconstruction may elicit the presence of a malignancy at the EGJ or significant fibrosis suggesting scar tissue formation from prior hiatal surgery. Endosonography (EUS) can also help discern any extrinsic compression at the EGJ in cases of pseudoachalasia, where the EUS does not demonstrate any pathology in patients with achalasia except for mucosal hypertrophy [9]. Additionally, inhalation of amyl nitrite, which allows the LES to relax and open in patients with achalasia but not in pseudoachalasia, can also be performed to distinguish between the two entities [10, 11]. Other diagnostic tests such as timed barium swallow are unreliable in distinguishing the two. And finally, while pneumatic dilatation or botox injection of the EGJ provides temporary relief in patients with achalasia, they do not affect the LES tone in patients with pseudoachalasia and can also be used as diagnostic tools.

Our patient had dysphagia almost 2 decades after his Nissen fundoplication and his endoscopy and esophagram were suggestive of achalasia. Several clinical features suggested otherwise. First, careful interpretation of the manometry clearly indicated normal relaxation of the LES and IRP [12]. In addition, at the time of the revisional surgery, the endoscope met considerable resistance at the EGJ, another feature inconsistent with primary achalasia [1]. During the laparoscopic dissection, we discovered that his fundoplication wrap had resulted in esophageal torsion, constricting the distal esophagus and confirming the diagnosis of pseudoachalasia. Once we took down the original fundoplication, corrected the torsion, and replaced it with a Dor partial fundoplication, he was able to resume normal feeding without further regurgitation. As suggested by Poulin et al., our particular case was most likely a type 2 pseudoachalasia, which is due to extensive development of scar tissue and/or tight fundic wrap that improves with removal of scar tissue and reconstruction of the wrap [3].

## Conclusions

Patients who undergo foregut surgeries can develop outflow obstruction that can mimic primary achalasia. Our case highlights the importance of an aggressive evaluation and careful interpretation of preoperative studies in any patient presenting with dysphagia after a remote history of fundoplication. These patients should be referred for surgery as appropriately indicated, and surgeons should then formulate a surgical plan after carefully reviewing the upper endoscopy and high-resolution manometry to determine alternative causes of outflow obstruction.

## Abbreviations

EGD, esophagogastroduodenoscopy; EUS, esophageal ultrasound; LES, lower esophageal sphincter; EGJ, esophagogastric junction; HRM, high-resolution manometry; IRP, integrated relaxation pressure; UGI, upper gastrointestinal series
